# Experimental Hyalohyphomycosis by *Purpureocillium lilacinum:* Outcome of the Infection in C57BL/6 Murine Models

**DOI:** 10.3389/fmicb.2017.01617

**Published:** 2017-08-23

**Authors:** Danielly C. M. de Sequeira, Rodrigo C. Menezes, Manoel M. E. Oliveira, Paulo R. Z. Antas, Paula M. De Luca, Joseli de Oliveira-Ferreira, Cintia de Moraes Borba

**Affiliations:** ^1^Laboratory of Taxonomy, Biochemistry and Bioprospecting of Fungi, Oswaldo Cruz Institute, Oswaldo Cruz Foundation Rio de Janeiro, Brazil; ^2^Laboratory of Immunoparasitology, Oswaldo Cruz Institute, Oswaldo Cruz Foundation Rio de Janeiro, Brazil; ^3^Laboratory of Clinical Research in Dermatozoonosis, Evandro Chagas National Institute of Infectology, Oswaldo Cruz Foundation Rio de Janeiro, Brazil; ^4^Laboratory of Mycology, Evandro Chagas National Institute of Infectology, Oswaldo Cruz Foundation Rio de Janeiro, Brazil; ^5^Laboratory of Clinical Immunology, Oswaldo Cruz Institute, Oswaldo Cruz Foundation Rio de Janeiro, Brazil

**Keywords:** *Purpureocillium lilacinum*, hyalohyphomycosis, experimental model, immune response, immunosuppression

## Abstract

*Purpureocillium lilacinum* is a filamentous, hyaline fungus considered an emerging pathogen in humans. The aim of our study was to evaluate the outcome of hyalohyphomycosis in C57BL/6 murine models inoculated with two clinical *P. lilacinum* isolates (S1 and S2). Each isolate was inoculated in mice randomly distributed in immunocompetent (CPT) and immunosuppressed (SPS) groups. Mice were evaluated at day 7, 21, and 45 after inoculation for histopathological analysis, recovery of fungal cells, and immunological studies. Histological analysis showed scarce conidia-like structures in lung tissue from CPT mice and a lot of fungal cells in SPS mice inoculated with S2 compared to mice inoculated with S1. The maximum recovery of fungal cells was seen in CPT mice inoculated with both isolates at day 7, but with mean significantly higher in those inoculated with S2 isolate. Phenotypical characterization of T cells showed TCD8^+^ lymphocytes predominance over TCD4^+^ in immunosuppressed mice infected and control groups. We also observed higher percentages of the central and effector memory/effector phenotype in CPT mice infected with S2 strain, especially in TCD8^+^ in the initial period of infection. Regulatory T cells showed higher percentages in immunosuppressed, predominantly after the acute phase. Our results showed that the *P. lilacinum* is a fungus capable to cause damages in competent and immunosuppressed experimental hosts. Furthermore, S2 isolate seems to cause more damage to the experimental host and it was possible to identify different cellular subsets involved in the mice immune response.

## Introduction

*Purpureocillium lilacinum* (Thom) Luangsa-ard, Houbraken, Hywel-Jones and Samson, comb. nov. is a filamentous, hyaline, anamorphic fungus (Luangsa-Ard et al., 2011) considered an emerging pathogen in humans, especially immunosuppressed. However, the number of immunocompetent hosts infected by this fungus increased in recent years (Saghrouni et al., 2013; Shivaprasad et al., 2013; Todokoro et al., 2014; Borba and Brito, 2016).

The clinical manifestations of hyalohyphomycosis caused by *P. lilacinum* may vary from localized infection in immunocompetent hosts to disseminated infection in immunosuppressed individuals. Infection occurs frequently due to trauma or implantation of surgical prosthetic devices, mainly intraocular lenses (Pastor and Guarro, 2006).

The virulence of *P. lilacinum* has been considered moderate by some authors and in experimental models it has been reported as low and requiring high inocula along with immunosuppression of the animals to establish infection (Pujol et al., 2002; Pastor and Guarro, 2006). However, our group showed the ability of this fungus to infect and cause disease in immunocompetent and immunosuppressed BALB/c mice with low inoculums (Brito et al., 2011).

Casadevall and Pirofski (2015) reported that infection is a necessary pre-condition for virulence, whereby virulence is the amount and degree of host damage stemming from host–microbe interaction. They also considered that damage and disease depend on the immune status of the host in addition to microbial and environmental factors.

Most data regarding the immune response show that CD4^+^ T cells are required for optimal host defense against several opportunistic pathogens. The differentiation of naive TCD4^+^ cells into specific helper T cells is influenced by the presentation of antigens by APCs and coupling of costimulatory molecules and by cytokines produced in the early stage of response to the antigen (Kaiko et al., 2008). In this context, regulatory T cells (Tregs), a subpopulation of specialized T lymphocytes, characterized by CD4^+^CD25^+^FoxP3^+^ phenotype, are responsible for maintaining homeostasis, by suppression, activation, proliferation, and effector function, as well as the production of cytokines from various cells with ability to control the immune response (Sakaguchi et al., 2010; Bacher et al., 2014; Schulze et al., 2014).

Despite the central role of TCD4^+^ lymphocytes in immunity against fungal infections, there is evidence that TCD8^+^ lymphocytes may also mediate a protective response against fungi, especially in cases of failures in the effector function of TCD4^+^, such as in immunosuppressive conditions (Vecchiarelli et al., 2007; Nanjappa et al., 2012a,b).

Immunological memory is critical for long-term immunity and protection from infection. After naive T cells are activated by the antigen, they differentiate into effector T cells which display different functions, depending on the anatomical position and phenotypic characteristics. However, only a small fraction of effector T cells becomes long-lived memory T cells to provide lifelong protection against the previously encountered pathogen (Sallusto et al., 2004). Their cell subtypes are defined by the expression of homing receptors CCR7 and CD62L according to their properties and categorized in central memory (T_CM_), effector memory (T_EM_), and resident memory (T_RM_) (Rivino et al., 2004). In fungal infections, in spite of the importance of TCD4^+^ memory cells, studies demonstrate that in the absence of these cells, TCD8^+^ effector lymphocytes can mediate resistance to fungi. These cells can differentiate into long-term memory cells and remain in circulation even in the absence of TCD4^+^ help, as well as retaining the ability to produce cytokines (Lindell et al., 2005; Nanjappa et al., 2012b).

Studies using animal models may demonstrate how fungi invade human bodies, the conditions that change fungal morphology and physiology, as well as an understanding the real mechanism of host defense (Pujol et al., 2002; Brito et al., 2011). Our group, exploring the *in vitro* interaction between peritoneal macrophages from C57BL/6 mice and *P. lilacinum* isolates, showed that conidia adhered to mammal cells were internalized and produced germ tube and mycelium (Peixoto et al., 2010). These results were significant in the choice of the *in vivo* C57BL/6 model with the same genetic lineage of the cells previously used, because they pointed out the possibility of disease establishment in these mice. The aim of this study was to evaluate the outcome of hyalohyphomycosis in immunocompetent and immunosuppressed C57BL/6 murine models inoculated with *P. lilacinum* clinical isolates and to characterize phenotypically the systemic immune response.

## Materials and Methods

### Animals

Inbred C57BL/6 specific pathogen free male mice, aged 6–8 weeks, weighting approximately 21 g, were maintained in facility of the Laboratory Animal Breeding Center (CECAL, FIOCRUZ) with individually ventilated cages and HEPA filtering system. Breeding room was managed at room temperature; 23–25°C, humidity; 30–70%, light/dark cycle; 12 h, and water and food were given *ad libitum*. This study was performed in strict accordance with the Brazilian College of Animal Experimentation (COBEA) and was performed under anesthesia, and all efforts were made to minimize the suffering of the animals. The responsible authority (Ethics Committee for Animal Study of FIOCRUZ; Permit Number L-041/07 - CEUA/FIOCRUZ) approved the study.

### Immunosuppressive Treatments

To perform immunosuppression mice received 5 mg/kg of dexamethasone (Hypofarma, Ribeirão das Neves, Brazil) administered *ad libitum* in the drinking water for 3 days before fungal inoculation and during all the experiments (Brito et al., 2011). Tetracycline (1000 mg/L, Teuto, São Paulo, Brazil) was also added to the drinking water in parallel in order to prevent bacterial infections.

### Fungal Isolates

Two human isolates of *P. lilacinum* isolated from subcutaneous lesions were used in this study: S1, kindly provided by Dr. Oliver Kurzai (Institut für Hygiene und Mikrobiologie, Würzburg, Germany) (Kurzai et al., 2003) and S2, provided by Dr. Annette Fothergill (Fungus Testing Laboratory, University of Texas Health Science Center, San Antonio, TX, United States).

### Culture Conditions

The isolates were subcultured in 50 ml of potato dextrose broth in a rotatory shaker at 100 rpm, for 7 days, at room temperature to evaluate the ability of sporulation of them. The fungal growth was incubated for 5 min at 4°C, homogenized by vortexing for 3 min and placed at 37°C for 5 min. The suspension was then centrifuged at 380 × *g* for 30 min, the number of conidia in the resultant supernatant (rich in conidia but free of hyphae) was quantified in a Neubauer hemocytometer and the pellet (mycelia mat) was weighted. The inoculum was prepared subculturing the isolates on potato dextrose agar (PDA) medium for 12 days, at room temperature. Then the conidia were collected by scraping the colonies, suspended in 50 mM phosphate-buffered saline (PBS) at pH 7.2 and the same thermal chock protocol described above was done. The viability of conidia was assessed by colony-forming unit (CFU) (Goihman-Yahr et al., 1980). Morphology analysis was done microculturing (Ridell, 1950) the isolates on PDA for 7 days, at room temperature. The morphology was observed after staining with Amann lactophenol plus cotton blue and examined under a Nikon light microscope, E400 model (Nikon Instruments Inc., Melville, NY, United States).

### Molecular Authentication and Reactivation of the Isolates

Genomic DNA was extracted from both isolates S1 and S2 to perform the molecular authentication at the species level. Partial sequencing of the internal transcribed spacer (ITS) region was evaluated using ITS5 (GGAAGTAAAAGTCGTAACAAGG) and ITS4 (TCCTCCGCTTATTGATATGC) (White et al., 1990). Briefly, the conditions were 100 ng DNA, 10 pmol of each primer, and an annealing temperature of 48°C. Automated sequencing was evaluated using the Sequencing Platform at Oswaldo Cruz Foundation PDTIS/FIOCRUZ, Brazil. The sequences were edited using Sequencer 4.9 software, compared using the BLAST and deposited in GenBank.

Three mice were inoculated with each isolate in order to reactive them. After 7 days, the fungal cells were recovered of spleen, grown in PDA medium and used to the experimental hyalohyphomycosis.

### Experimental Hyalohyphomycosis

Each fungal isolate was inoculated in 60 mice randomly distributed in two groups: 30 immunocompetent (CPT) and 30 immunosuppressed mice (SPS). Thirty mice were similarly distributed as 15 CPT and 15 SPS control groups and inoculated with PBS. After administration of anesthetic eye drops with 0.5% proparacaine hydrochloride (Alcon, Rio Pequeno, Brazil), mice were inoculated intravenously (i.v.) into the right retro-orbital plexus, as previously described (Borba et al., 2005) with 0.02 ml of sterile PBS containing 1 × 10^5^ conidia with more than 80% viability. The control groups were similarly inoculated with sterile PBS. This route was chosen to be less traumatic and safer than a tail vein (Steel et al., 2008). Mice were checked daily for 45 days to observe weight loss and disease symptoms. They were anesthetized and sacrificed by CO_2_ exposure at 7, 21, and 45 days after inoculation or when they presented cruel signs of suffering and used for different analysis. The experimental hyalohyphomycosis was done in duplicate to ensure reproducibility.

### Immune Response Evaluation

Twenty-four mice (12 CPT and 12 SPS) were inoculated with S2 isolate and 16 inoculated with PBS (eight CPT and eight SPS), distributed in groups as described above in order to evaluate cellular immune response. Mice were euthanized 7 and 14 days after inoculation for flow cytometric analysis and these experiments were also done twice to ensure reproducibility.

### Blood Collection, Necropsy, and Histopathology

Mice were weighted, submitted to anesthesia with 10% ketamine hydrochloride associated to 2% xylazine hydrochloride (Syntec, Santana de Parnaíba, Brazil). Blood samples collection was performed by cardiac puncture. After animal euthanasia, spleen, lungs, and liver were removed at days described in experimental hyalohyphomycosis item. The lung and liver specimens obtained from mice were fixed in 10% neutral buffered formalin, dehydrated, and embedded in paraffin. Sections were cut and stained with hematoxylin and eosin, periodic acid-Schiff (PAS), and Grocott’s (methenamine silver) and the photographs were taken by Leica DM 1000 (Leica, Wetzlar, Germany).

### Recovery of Fungal Cells

Spleens and fragments of liver and lungs were aseptically removed and homogenized in sterile complete RPMI-1640 medium containing L-glutamine (Sigma Chemical, St Louis, MO, United States) to determine the number of CFU. The suspension was adjusted to 2 mg of tissue per ml and samples of 150 μl of each homogenate were transferred to Petri dishes with Mycosel agar (Becton Dickinson and Company, Sparks, MD, United States) and incubated at 25°C for 60 days for fungal re-isolation.

### Flow Cytometry Analysis

Splenic cells recovered from mice at days 7 and 21 after inoculation were suspended in RPMI-1640 medium after removing the red blood cells with RBC lysis buffer (0.1 M NH_4_Cl, pH 7.15). After three washings with cold staining buffer (PBS, 0.1% BSA, 0.01% sodium azide, all from Sigma-Aldrich, United States), cells (10^5^/50 μl) were harvested and stained with CD4-FITC and CD8-PE monoclonal antibodies. Thereafter the cells were kept in the dark for 30 min at 4°C. After staining, the cells were washed with PBS, and these cells were fixed using 1% formaldehyde solution (Sigma-Aldrich) until acquisition. To investigate the generation of memory T cells was used the same protocol, however, cells were harvested and stained with CD3-Alexa 647, CD4-APCCy7, CD8-PECF594, CD44-V500, CD62L-Alexa 700, and CD197(CCR7)-PerCP5.5. To evaluate the presence of Tregs, in addition to extracellular labeling using CD4-FITC and CD25-PECy7, intranuclear labeling was performed with FoxP3-APC. All monoclonal antibodies were produced in rat and were acquired from Becton Dickinson and Company (Beckman Coulter, Brea, CA, United States). All experiments were carried out using a Gallios flow cytometer device (Beckman Coulter, Brea, CA, United States). A minimum of 10,000 events per sample were acquired inside the lymphocytes gate, based on size and granularity properties and analyzed using FlowJo (Flow Cytometry Analyses Software, Tree Star, Ashland, OR, United States).

### Statistical Analysis

Data were analyzed using GraphPad Prism^®^ software, version 5 (GraphPad Software, Inc., San Diego, CA, United States). Data are expressed as mean ± standard deviation. Mann–Whitney test was used to perform comparisons between groups in CFU and immunological experiments. A *p*-value of 0.05 or less was considered significant.

## Results

### Fungal Morphology and Molecular Authentication

**FIGURE 1:**
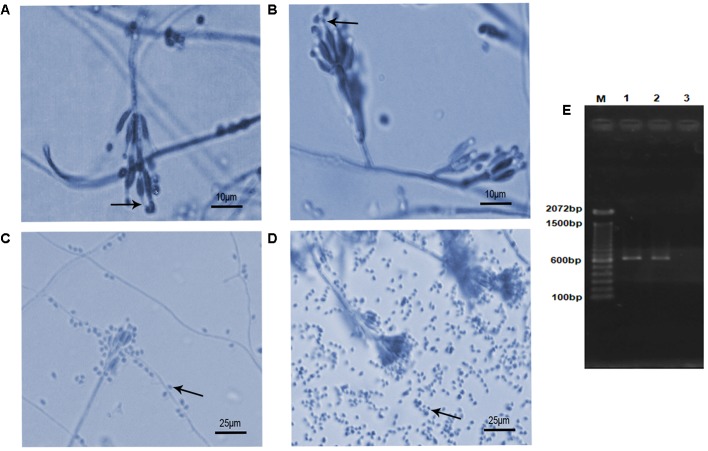
Microscopic morphology and molecular authentication of *Purpureocillium lilacinum* isolates. **(A–D)** Lactophenol-cotton-blue stained preparation of a 7-day-old culture on potato dextrose agar medium. **(A)** S1 isolate showing typical phialides with a distinct neck (arrow) bearing ellipsoid conidia; **(B)** S2 isolate showing the same typical structures (arrow) seen in panel **(A)**; **(C)** conidia (arrow) produced by S1 isolate; **(D)** conidia (arrow) produced by S2 isolate; **(E)** PCR product based on the partial internal transcribed spacer (ITS) region with ITS4–ITS5 primers pair. M, molecular marker DNA ladder, 100 bp (Invitrogen); 1, S1; 2, S2; 3, negative control.

The isolates presented morphological characteristics consistent with those described in the literature (Luangsa-Ard et al., 2011), that is, colony white at first, becoming vinaceous; reverse in shades of purple. Conidiophores verticillate with two or four phialides having a swollen basal portion tapering into a short distinct neck (**Figures 1A,B**). Conidia hyaline, ellipsoidal to fusiform. Chlamydospores absent. However, they showed differences in the ability to produce conidia. The **Table 1** shows the number of conidia and the mycelial mass weigh produced for both isolates. The isolate S2 produced a substantial amount of mycelium and more conidia (**Figure 1D**) than S1 isolate (**Figure 1C**) at the 7th day of growth.

**Table 1:** Quantification of mycelium mat and conidia produced by *Purpureocillium lilacinum* isolates (S1 and S2) in potato dextrose broth.

Isolate	Dry weight of mycelium (g)	Number of conidia/ml
S1	3.31	3.6 × 10^6^
S2	11.89	1.0 × 10^7^

The BLAST analysis comparing the ITS sequences obtained from PCR product of S1 and S2 isolates (**Figure 1E**) with sequences deposited in the NCBI GenBank database (S1- MF590108; S2- MF590109) allowed to identify these isolates as *P. lilacinum* with 99–100% similarity with other *P. lilacinum* sequences (KY630747, KU196096, KY786077, KU747154, KT224843, KC403967).

### Clinical Manifestations of Infected Mice

**FIGURE 2:**
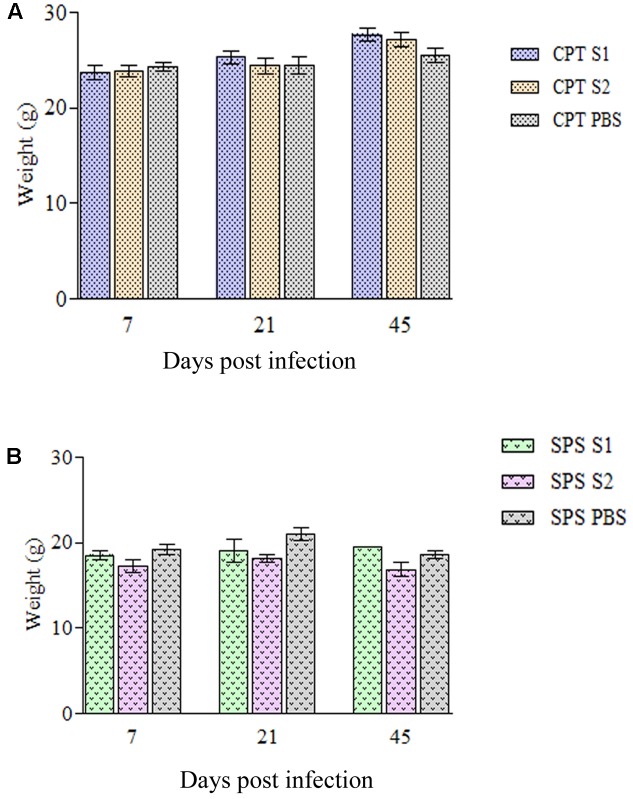
Weigh variation of immunocompetent (CPT) and immunosuppressed (SPS) mice inoculated with *Purpureocillium lilacinum* isolates. **(A)** Weight variation in immunocompetent mice inoculated with both S1 and S2 isolates and control group inoculated with PBS; **(B)** weight variation in immunosuppressed mice inoculated with both S1 and S2 isolates and control group inoculated with PBS. Bars represent the mean of weight variation in mice. CPT S1, immunocompetent mice inoculated with S1 isolate; CPT S2, immunocompetent mice inoculated with S2 isolate; CPT PBS, immunocompetent mice inoculated with PBS; SPS S1, immunosuppressed mice inoculated with S1 isolate; SPS S2, immunosuppressed mice inoculated with S2 isolate; SPS PBS, immunosuppressed mice inoculated with PBS.

Immunocompetent mice inoculated with both *P. lilacinum* isolates (S1 and S2) showed no clinical signs of the disease except one CPT mouse inoculated with S1 isolate, at day 45 postinfection which presented severe keratitis on right eye with positive culture to *P. lilacinum* (data not shown). All the other CPT animals were active and had weigh gain (**Figure 2A**) throughout the observed period (45 days). In contrast, SPS mice inoculated with both isolates including control group, became lethargic and had weight (**Figure 2B**) and hair loss. Moreover, in SPS infected mice, both isolates were capable to cause tail necrosis at day 21 postinfection and those inoculated with S2 presented dermatitis and abdominal nodules. In animals with lesions, fungal cells were recovered from skin.

### Histological Studies

**FIGURE 3:**
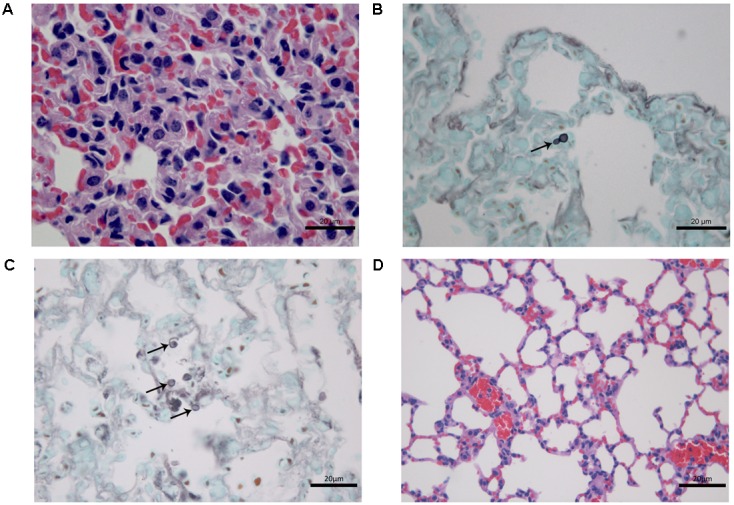
Histological section of tissues of immunocompetent (CPT) mice inoculated with *Purpureocillium lilacinum* isolates at 7 days after infection. **(A)** Lung tissue of CPT mouse infected with S1 isolate: pneumonia with inflammatory infiltrate composed of macrophages and a few neutrophils, H&E; **(B)** a budding yeast-like cell (arrow) is seen in lung tissue of CPT mouse infected with S1 isolate, Grocott; **(C)** lung tissue of CPT mouse infected with S2 isolate: conidia-like structures were seen in the alveolar wall (arrows), Grocott; **(D)** lung tissue of CPT control mouse inoculated with PBS, H&E.

At 7 days after infection, histopathological study of lung tissues of CPT mice inoculated with S1 isolate showed a granulomatous multifocal and moderate pneumonia. The inflammatory infiltrate was composed of macrophages and a few neutrophils (**Figure 3A**) and were also observed budding yeast-like cells (**Figure 3B**). Mice inoculated with S2 isolate showed no lesions, but conidia-like structures were seen in the alveolar wall (**Figure 3C**). No histological changes were observed in the hepatic tissue of CPT mice infected with both isolates. Lung (**Figure 3D**) and liver tissues of CPT control mice did not show histological alterations. With respect to SPS groups, were not observed histopathological alterations and fungal cells in lung and liver tissues of the mice inoculated with both isolates.

**FIGURE 4:**
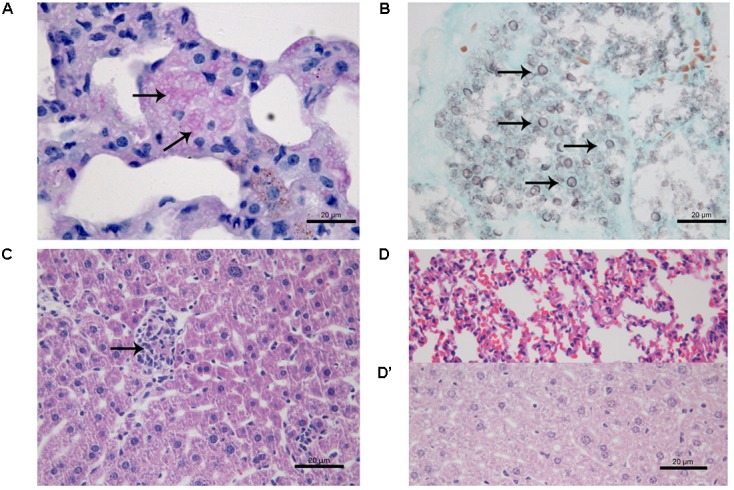
Histological section of tissues of immunocompetent (CPT) mice inoculated with *Purpureocillium lilacinum* S2 isolate **(A–C)** and control group inoculated with PBS **(D,D’)** at 21 days after infection. **(A)** Lung tissue shows pneumonia with moderate inflammatory infiltrate composed of macrophages and neutrophils (arrows), H&E; **(B)** conidia-like structures (arrows) are seen in lung tissue, Grocott; **(C)** mild inflammatory infiltrate in the hepatic parenchyma (arrow), H&E; **(D)** lung and **(D’)** liver tissues of CPT control mice without histological alterations, H&E.

**FIGURE 5:**
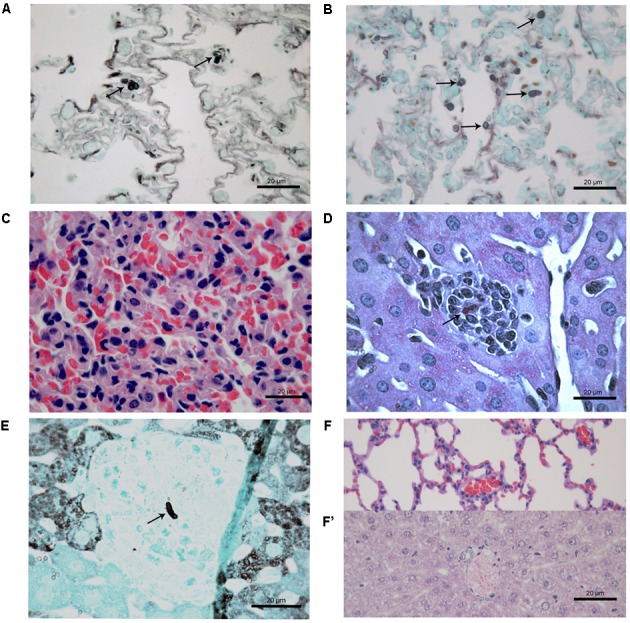
Histological section of tissues of immunosuppressed (SPS) mice inoculated with *Purpureocillium lilacinum* isolates **(A–C)** and control group inoculated with PBS **(F,F’)** at 21 days after infection. **(A)** Conidia-like structures inside macrophages in the lung tissues of SPS mice infected with S1 isolate (arrows), Grocott; **(B)** lung tissue of SPS mice infected with S2 isolate showing conidia-like structures (arrows), Grocott; **(C)** multifocal pneumonitis, with moderate inflammatory infiltrate composed of macrophages and neutrophils in lung tissue of SPS mice infected with S2 isolate; **(D)** liver tissue of SPS mice infected with S1 isolate showing hepatitis with multifocal and moderate inflammatory infiltrate composed predominantly of macrophages and neutrophils with conidia-like structure (arrow), H&E; **(E)** tissue of SPS mice infected with S1 isolate with conidia-like structure in inflammatory infiltrate in regions of the liver parenchyma (arrow), Grocott; **(F)** lung and **(F’)** liver of SPS control mice without histological alterations, H&E.

At 21 days after infection, CPT mice inoculated with S1 isolate did not present histological changes and fungal cells in liver and lung tissues. In contrast, CPT mice inoculated with S2 isolate presented multifocal pneumonia, with moderate inflammatory infiltrate composed of macrophages and neutrophils (**Figure 4A**), and conidia-like structures in the lung tissues (**Figure 4B**). Mild inflammatory infiltrate was also observed in the hepatic parenchyma of mice of this group (**Figure 4C**). No histological changes were observed in lung and hepatic tissues of CPT control mice (**Figures 4D,D’**). In the SPS mice inoculated with S1 isolate it was not observed inflammatory infiltrate in the lung tissues, in spite of the presence of conidia-like structures inside macrophages (**Figure 5A**). SPS mice inoculated with S2 isolate presented conidia-like structures in the lung tissues (**Figure 5B**), multifocal pneumonitis, with moderate inflammatory infiltrate composed of macrophages and neutrophils (**Figure 5C**). In the liver tissue, only SPS mice inoculated with S1 isolate presented hepatitis with multifocal and moderate inflammatory infiltrate composed predominantly of macrophages and neutrophils (**Figure 5D**) and conidia-like structures were observed in the inflammatory infiltrate in regions of the liver parenchyma (**Figure 5E**). SPS control mice did not show histological alterations in lung (**Figure 5F**) and liver (**Figure 5F’**) tissues.

**FIGURE 6:**
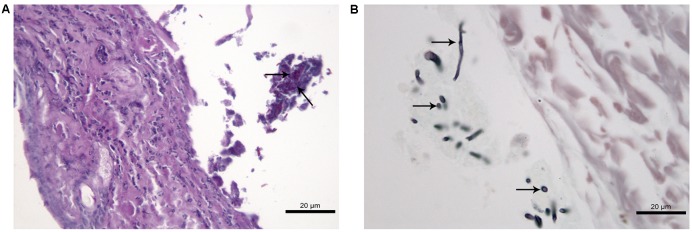
Histological section of cutaneous tissue of immunosuppressed (SPS) mice inoculated with *Purpureocillium lilacinum* S2 isolate at 45 days after infection. **(A)** Dermatitis with diffuse inflammatory infiltrate formed by neutrophils and an abscess in the subcutaneous tissue with conidia and hyphae-like fragments (arrows), PAS; **(B)** conidia and hypha-like structures are seen within the abscess (arrows), Grocott.

At 45 days after infection, no fungal cells and histological changes were observed in tissues of CPT mice inoculated with both isolates. SPS mice inoculated with S1 isolate presented yeast-like cells in lung tissues but without inflammatory infiltrate while SPS mice inoculated with S2 isolate presented a multifocal and mild lympho-histiocytic hepatitis but without fungal structures. Histological analysis of skin nodules from abdominal region of SPS mice inoculated with S2 isolate, 45 days after infection, showed dermatitis with diffuse inflammatory infiltrate composed of neutrophils and an abscess in the subcutaneous tissue (**Figure 6A**). Conidia and hyphae-like fragments were observed within the abscess in the subcutaneous tissue (**Figure 6B**), with culture positive to *P. lilacinum*. These nodules were not observed in mice inoculated with S1 isolate.

### Colony-Forming Unit or Fungal Cells Recovered

**FIGURE 7:**
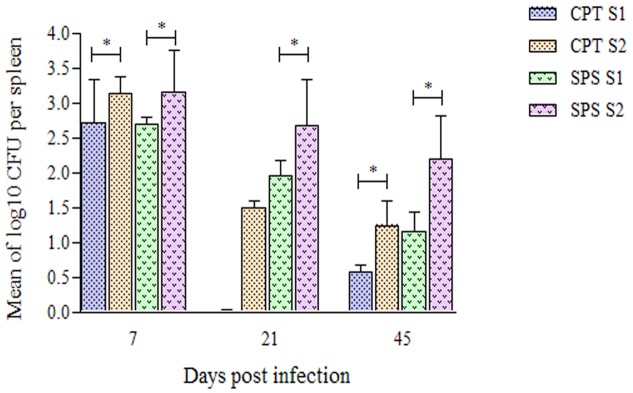
Number of *Purpureocillium lilacinum* cells recovered from spleen of immunocompetent (CPT) and immunosuppressed (SPS) mice inoculated with both S1 and S2 isolates. Bars represent the mean of colony-forming units recovered from spleen of six mice euthanized 7, 21, and 45 days after inoculation. Data are representative of two independent experiments with *n* = 6 infected mice in each experiment. Unpaired Mann–Whitney *U*-test was utilized to determine significant differences ^∗^*P* < 0.05.

Fungal cells were recovered from spleen of CPT and SPS mice on days 7, 21, and 45 postinoculation (**Figure 7**). At day 7 fungal cells were recovered in higher numbers in mice inoculated with both isolates. In contrast, in CPT mice inoculated with S1 isolate, fungal cells were not recovered at day 21 postinoculation. However, fungal cells were recovered in this group at day 45 postinoculation. CPT and SPS mice inoculated with S2 isolate presented fungal cell recovery, at all time analyzed, with recovering peak at day 7, followed by a decrease at days 21 and 45 postinoculation. Furthermore, the mean number of fungal cells recovered from the spleen of mice inoculated with S2 was significantly higher (*P* < 0.05) than that from those inoculated with S1 isolate in both murine models (CPT and SPS). No fungal cells were recovered from liver and lungs at all points and in both CPT and SPS mice.

### CD4 and CD8 T Cells Quantification

**FIGURE 8:**
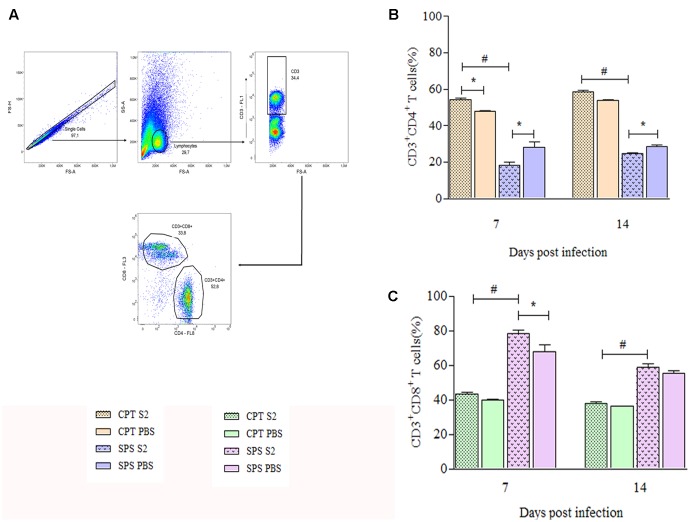
Quantification of CD4^+^ and CD8^+^ T cells recovered from spleens of immunocompetent (CPT) and immunosuppressed (SPS) mice inoculated with *Purpureocillium lilacinum* and control groups at days 7 and 14 postinoculation. **(A)** Dot-plot representing gating strategy. Gating of total events included a singlet cell gate; followed by selection for CD3^+^ T cells inside the lymphocytes gate. CD4^+^ and CD8^+^ T cells were identified by CD3^+^ and CD4^+^ or CD8^+^ expression; **(B)** percentage of TCD4^+^ cells of immunocompetent and immunosuppressed mice infected with S2 isolate and PBS inoculated control groups at 7 and 14 days after infection; **(C)** percentage of CD8^+^ T cells from immunocompetent and immunosuppressed mice infected with S2 isolate and PBS inoculated control groups at 7 and 14 days after infection. CPT S2, immunocompetent mice inoculated with S2 isolate; CPT PBS, immunocompetent mice inoculated with PBS; SPS S2, immunosuppressed mice inoculated with S2 isolate; SPS PBS, immunosuppressed mice inoculated with PBS. Data are representative of two independent experiments with *n* = 6 infected mice and *n* = 4 control mice in each experiment. Unpaired Mann–Whitney *U*-test was utilized to determine significant differences ^∗#^*P* < 0.05.

Since our results suggested that S2 isolate was more aggressive than S1, we performed experiments to analyze the cellular immune response against this isolate. Phenotypical characterization of T cells in total splenocytes was performed by flow cytometry at days 7 and 14 postinfection. First, were gated TCD4^+^ and TCD8^+^ lymphocytes based on size and granularity (**Figure 8A**) and once these populations were selected, the quantification of the cells in the CPT and SPS groups was performed. Throughout the experimental period, CPT group presented significant higher percentages of CD4^+^ T lymphocytes (*P* < 0.05), especially in the infected group, than SPS mice. The SPS mice, unlike CPT mice, presented even lower percentages of TCD4^+^ in the infected group (**Figure 8B**). Analyzing the percentages of TCD8^+^ in CPT groups at 7 and 14 days after infection, as expected, CPT groups presented lower percentages of CD8^+^ T cells in relation to CD4^+^ T cells, but the percentages of CD8^+^ T cells were higher in infected group in comparison to their control. However, in SPS mice the percentage of CD8^+^ T cells was higher in infected mice group when compared to CPT group (*P* < 0.05) over the observation period. In mice of the SPS control group the percentage of CD8^+^ T cells was also higher in the beginning of the infection (7 days) (**Figure 8C**).

### Generation of Tregs and Memory T cells

**FIGURE 9:**
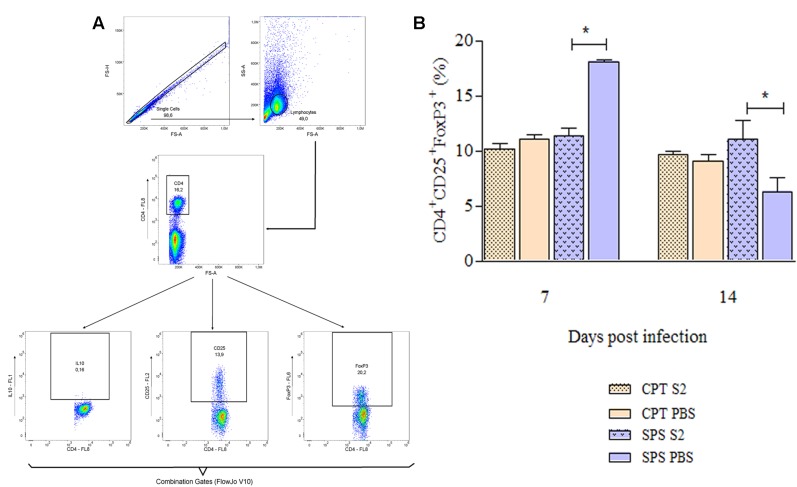
Expression of regulatory T cells markers of mice inoculated with *Purpureocillium lilacinum* and control groups at days 7 and 14 postinoculation. **(A)** Dot-plot representing gating strategy. Gating of total events included a singlet cell gate; followed by selection for CD4^+^ T cells inside the lymphocytes gate. CD25^+^and FoxP3^+^ cells were identified in CD4^+^ T lymphocytes. Boolean combinations were used to the two total gates (CD25 and FoxP3) to uniquely discriminate T regulatory cells population; **(B)** percentage of CD4^+^CD25^+^FoxP3^+^ regulatory T cells in the spleens of immunocompetent and immunosuppressed mice infected with S2 isolate and inoculated with PBS (control groups), at 7 and 14 days after infection. CPT S2, immunocompetent mice inoculated with S2 isolate; CPT PBS, immunocompetent mice inoculated with PBS; SPS S2, immunosuppressed mice inoculated with S2 isolate; SPS PBS, immunosuppressed mice inoculated with PBS. Data are representative of two independent experiments with *n* = 6 infected mice and *n* = 4 control mice in each experiment. Unpaired Mann–Whitney *U*-test was utilized to determine significant differences ^∗^*P* < 0.05.

**FIGURE 10:**
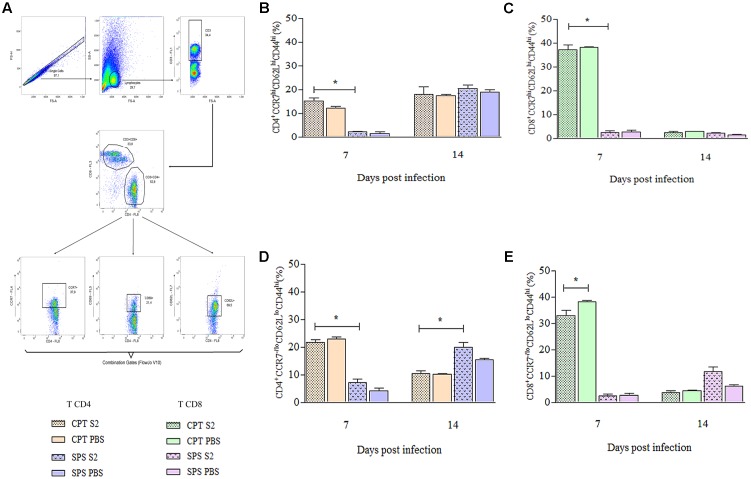
Expression of memory markers on TCD4^+^ and TCD8^+^ lymphocytes collected from spleen of mice inoculated with *Purpureocillium lilacinum* S2 isolate and control groups at days 7 and 14 postinoculation. **(A)** Dot-plot representing gating strategy. Gating of total events included a singlet cell gate; followed by selection for CD3^+^ T cells inside the lymphocytes gate. CD4^+^ and CD8^+^ T cells were identified by CD3 and CD4 or CD3 and CD8 expression. CD44^+^, CD62L^+^, and CCR7^+^ T cells were identified in CD4^+^ or CD8^+^ T cell populations. Boolean combinations were used to the three total gates (CD44, CD62L, and CCR7) to uniquely discriminate memory CD4^+^ and CD8^+^ T cells populations; **(B–E)** percentage of central memory (CD44^+^CD62L^+^CCR7^+^) and effector/effector memory (CD44^+^CD62L^-^CCR7^-^) T cells: **(B)** CD4^+^ T central memory; **(C)** CD8^+^ T central memory; **(D)** CD4^+^ T effector/effector memory; **(E)** CD8^+^ T effector/effector memory. CPT S2, immunocompetent mice inoculated with S2 isolate; CPT PBS, immunocompetent mice inoculated with PBS; SPS S2, immunosuppressed mice inoculated with S2 isolate; SPS PBS, immunosuppressed mice inoculated with PBS. Data are representative of two independent experiments with *n* = 6 infected mice and *n* = 4 control mice in each experiment. Unpaired Mann–Whitney *U*-test was utilized to determine significant differences ^∗^*P* < 0.05.

Flow cytometry was also used to identify T cells that express regulatory (CD4^+^CD25^+^FoxP3^+^), central (CD4^+^/CD8^+^CD44^+^CD62L^+^CCR7^+^) and effector/effector memory (CD4^+^/CD8^+^CD44^+^CD62L^-^CCR7^-^) phenotypes from mice infected with S2 isolate. These experiments were also performed at days 7 and 14 postinfection. Concerning Tregs was used boolean combination gates to select the TCD4^+^CD25^+^FoxP3^+^ population and perform the analysis (**Figure 9A**). CPT mice did not present statistical differences in the Treg expression between infected and control group over the observation period. However, infected SPS mice presented significant difference (*P* < 0.05) from its control (SPS PBS). At 7 days postinfection were observed higher percentages of Tregs in control group compared to infected group, reversing at 14 days postinfection, when the percentage of Tregs was higher in infected mice compared to control (**Figure 9B**). As for memory T cells, **Figure 10A** shows the gating strategy for analyzing central and effector/effector memory T cell phenotypes. Central memory CD4^+^ T cells from CPT mice presented significant difference (*P* < 0.05) when compared to SPS group at 7 days postinfection. However, at 14 days postinfection, no differences were observed among groups (**Figure 10B**). Similar results were observed for CD8^+^ central memory T cells at days 7 and 14 postinfection (**Figure 10C**). When quantifying TCD4^+^ and TCD8^+^ effector/effector memory lymphocytes (**Figures 10D,E**), our results indicated that CPT mice presented significant higher percentages (*P* < 0.05) of this memory phenotype when compared to SPS mice at 7 days after inoculation. However, at day 14 postinfection, effector/effector memory CD4^+^T and CD8^+^ T cells reversed this profile, that is, the cells collected from SPS mice presented greater expression of effector/effector memory markers.

## Discussion

Pathogenicity has been considered an inherent, genetic capacity of a microorganism to cause disease, mediated by specific virulence factors and virulence a property inherent to host–parasite relationship, once the same parasite can be virulent in a susceptible host and avirulent in another non-susceptible host (Casadevall and Pirofski, 1999). Some characteristics, as immunosuppression, increase the ability of a pathogen to infect a host, and according to Casadevall et al. (2011), infection is a condition for virulence. Our results corroborate those authors statement since experimental host immunosuppression was necessary to provoke hyalohyphomycosis clinical signs by *P. lilacinum* in C57BL/6 murine model as well as in BALB/c mice studied by Brito et al. (2011).

The concentration of conidia and inoculation route is very important to reproduce the infection and disease (Latge, 2001). Some authors described the necessity of a high inoculum—10^6^ to 10^8^ conidia—and immunosuppression to produce an established experimental infection with *P. lilacinum* (Pujol et al., 2002; Pastor and Guarro, 2006). However, Brito et al. (2011) using an inoculum of 10^4^ conidia were able to produce infection and disease in competent and immunosuppressed BALB/c models by the intravenous route similar to the infection caused in the C57BL/6 model used here. Although C57BL/6 mice are described as more resistant to some microorganisms, for example, *Leishmania* spp. (Sacks and Noben-Trauth, 2002) we obtained infection using an inoculum with 10^5^ viable conidia. In addition, our results showed that C57BL/6 murine model was more resistant than BALB/c used by our group with the same species (Brito et al., 2011). Disappointingly, studies using animal models for *P. lilacinum* are scarce and some of them did not describe the route of inoculation (Antas et al., 2011). Previous finding using immunocompetent ICR white mice inoculated with conidia by intraperitoneal route showed lesions in different organs, but the authors did not succeed in the recuperation of *P. lilacinum* isolates (Hubálek and Hornich, 1977). On the other hand, fungal cells were mainly recovered from immunosuppressed mice (albino and OF_1_ mice) inoculated using the routes intraperitoneal and lateral tail vein and were seen lesions in the liver, spleen, lungs, kidney, heart, brain, and eyes (Agrawal et al., 1979; Pujol et al., 2002) similar to lesions found in the models used here. More recently, Yuan et al. (2009) demonstrated *P. lilacinum* keratitis only in immunosuppressed BALB/c mice after inoculation of small inocula of 10^6^ conidia and 10^3^ hyphae by corneal scarification route, the same clinical manifestation seen in only one immunocompetent mouse used here. Notwithstanding the best route fungal infection is described, as the path would mimic the natural route (Latge, 2001) we elected the intravenous route to escape of innate immune response that might kill the fungus (Jiménez-López and Lorenz, 2013). Additionally, the retro-orbital plexus is reported as safe and effective site for injections and cause less stress to the mouse than lateral tail vein (Steel et al., 2008). Moreover, our group has been using this venous route with success (Borba et al., 2005; Brito et al., 2011).

The results of number of fungal cells recovered from spleen and histopathological analysis suggest greater ability of the S2 isolate to invade the experimental host than S1 isolate. The higher number of viable cells recovered from spleen for S2 isolate compared to S1 isolate was statistically significant in both experimental models, and a lot fungal cells were found in histological sections of lungs of SPS and CPT mice and abdominal nodules of SPS mice.

Furthermore, the human isolates studied here presented similar morphological characteristics, but the ability to produce conidia was different between them. The S2 isolate produced conidia *in vitro* earlier than the S1 isolate. The ability of the former isolate could make it capable of propagating faster inside the host since the ability to sporulate in infected tissue is a peculiar characteristic of *P. lilacinum* (Liu et al., 1998). *Fusarium* species as well as *P. lilacinum* form budding cells, phialides and phialoconidia within the infected tissue. These can enter the bloodstream and circulate within it more easily than hyphal structures (Perfect and Schell, 1996). Our data reinforce that phenotypical differences may promote a higher adaptation to the environment and increase the ability of the fungus to invade and colonize the host, as speculated by Brito et al. (2011) when comparing two *P. lilacinum* isolates from environmental and human sources. These authors using BALB/c mice suggested that the human isolate was more invasive than the environmental isolate. On the other hand, the C57BL/6 murine model used here showed that although both isolates were clinical they had different ability to invade the immunocompetent model.

Brito et al. (2011) affirmed that the optimum temperature for growth and sporulation of *P. lilacinum in vitro* ranged between 20 and 25°C and this characteristic might explain the presence of fungal cells and lesions at body extremities and only a transient presence of fungi in deep tissues of the BALB/c as well as C57BL/6 mice seen in this study. The same authors also observed corneal opacification of the immunosuppressed mice eyes different that was observed in C57BL/6, exception for one mouse with severe keratitis on right eye that could be dependent of the host condition.

Host conditions can influence the course of the disease (Casadevall and Pirofski, 2000). For this reason, the immune profile of immunocompetent and immunosuppressed mice used here was evaluated by quantifying the total number and the phenotype of splenocytes by flow cytometry. Our data showed the immunosuppressive action of dexamethasone, since immunosuppressed mice presented significant lower number of splenocytes, when compared to immunocompetent mice especially in infected mice. Morphological alterations and splenocyte death have been demonstrated after using dexamethasone as immunosuppressive agent besides weight loss in animal model as seen in our experiment (Miller and Schaefer, 2007).

It has been demonstrated in different systems that dexamethasone is capable of inhibiting the production of many cytokines, especially IL-2, interfering with the clonal expansion and differentiation of CD4^+^ and CD8^+^ T cells (Furue and Ishibashi, 1991; Paliogianni et al., 1993; Diasio and LoBuglio, 1996). CD4^+^ T lymphocytes are described as important protective cells in fungal infections (Romani, 2011). They produce IFN-γ, a cytokine associated to cell activation, and some authors affirm that depleting CD4^+^ T cells dramatically decreases IFN-γ production and accelerates mortality in mice. On the other hand, CD8^+^ T cell depletion or β2-microglobulin deficiency only marginally affects fungal clearance (Lin et al., 2005). However, Lindell et al. (2005) and Lin et al. (2005), studying cryptococcosis and histoplasmosis, respectively, showed that occasionally CD4^+^ T cells were not required for the CD8^+^ T cell-mediated immune response. These authors demonstrated importance and increase of CD8^+^ T cells in protecting mice lacking functional CD4^+^ T cells. Our results clearly demonstrated the predominance of CD8^+^ T cells over the mice groups with CD4^+^ T cells deficiency.

The predominance of CD8^+^ T cells in mice immunosuppressed with dexamethasone observed in our study is similar to the results of Miller and Schaefer (2007). The authors suggested that CD8^+^ T cell populations are resistant to the effects of dexamethasone and thus continue to proliferate while CD4^+^ T cells undergo apoptosis.

In our study, it was possible to identify the presence of Tregs, noting that at 7 days postinfection, CPT and SPS mice cells infected with the fungus had lower percentages of this phenotype, compared to the respective controls. This profile was also observed by Berod et al. (2014) in experimental infection of mice with *Mycobacterium bovis* and the authors concluded that the possible explanation for this fact is due to the higher production of effector T cells in the acute phase of the infection with the objective to contain the action of the pathogen. After this period, it was possible to notice in the present study that the infected groups started to express more Tregs than their controls, especially the immunosuppressed ones. Also, according to these authors, the higher percentages of this phenotype in infected SPS may be due to the fact that Tregs are more sensitive to the PAMPs recognized by TLR2. Therefore, a greater fungal load of SPS animals would explain this predominance of Tregs in relation to CPT.

Additionally, we could identify effector or central/effector memory in our study. We observed that at the beginning of the infection, the largest populations found were TCD4^+^/TCD8^+^ effector or central/effector memory lymphocytes from CPT mice, especially TCD8^+^. According to some authors, TCD8^+^ lymphocytes require a shorter time to initiate clonal expansion and, consequently, respond to antigenic stimulus (Shedlock and Shen, 2003; Ravkov and Williams, 2009). Further as regards the TCD8^+^ cells, in CPT group, this difference was greater in the control group, unlike the SPS, whose infected animals presented higher percentages of this phenotype, compared to those that were inoculated with PBS. We can suppose that, because of TCD4^+^ deficiency, cells of SPS mice have expressed this phenotype in an attempt to contain the fungal load (De Boer et al., 2003).

## Conclusion

In conclusion, it is clear that the *P. lilacinum* isolates from human can infect both immunocompetent and immunosuppressed C57BL/6 mice model. Furthermore, it was possible to suggest greater ability of the S2 isolate to cause more damage to the experimental host. Thus, we sought to explore the bases of cellular immune response of the host challenged with this fungal isolate, and it was possible to identify different cellular subsets involved in the response. We need to understand the mechanisms of fungal survival to host response and afterward to elucidate the complex mechanisms involved in the pathogenesis to improve the treatment schemes. For this reason, further studies using experimental model are necessary to get appropriate answers.

## Author Contributions

DCMS, JOF, and CMB designed the study. DCMS carried out experiments. DCMS, RCM, PMD, JOF, and CMB analyzed the data. DCMS and CMB wrote the manuscript. MMEO carried out molecular experiments. DCMS, RCM, MMEO, PRZA, PMD, JOF, and CMB revised the manuscript and all the authors approved the final manuscript.

## Conflict of Interest Statement

The authors declare that the research was conducted in the absence of any commercial or financial relationships that could be construed as a potential conflict of interest.
